# Microbubble-free mechanical sonoporation for MR-guided drug delivery in solid tumours: a proof-of-concept study

**DOI:** 10.1007/s13346-026-02078-w

**Published:** 2026-02-19

**Authors:** Deyssy Patrucco, Martina Capozza, Francesca Garello, Alberto Mangia, Carla Carrera, Giulia Tarso, Enzo Terreno

**Affiliations:** Molecular and Preclinical Imaging Centre, Department of Molecular Biotechnology and Health Sciences, University of Torino, Turin, Italy

**Keywords:** Sonoporation, Microbubble-free ultrasound, Drug delivery, MRI contrast agents, Intracellular uptake

## Abstract

Ultrasound (US)-mediated drug delivery is a promising strategy for localised, minimally invasive molecular transport, typically relying on microbubble-assisted sonoporation. However, clinical translation is limited by the short half-life, cost, and cytotoxicity of exogenous microbubbles. In this proof-of-concept study, we present a microbubble-free, mechanically driven sonoporation approach for intracellular delivery of hydrophilic agents, monitored non-invasively by magnetic resonance imaging (MRI). Using a low-intensity, non-focused ultrasound protocol (1 MHz, MI 0.25, 25% duty cycle, 1 Hz PRF) we achieved efficient intracellular internalisation of the MRI contrast agent Gadoteridol (~ 7 × 10⁹ Gd ions/cell) in vitro while maintaining > 70% cell viability and demonstrating complete membrane resealing within 30 min. Co-delivery with propidium iodide (PI) was confirmed by fluorescence microscopy and MRI, showing cytosolic distribution with negligible passive uptake in non-sonoporated controls. Compared to established permeabilisation methods (electroporation, pinocytosis, hypotonic swelling), microbubble-free sonoporation produced higher uptake with lower oxidative stress. In vivo experiments in K562 tumour-bearing mice showed that intravenous co-administration of Gadoteridol and PI followed by local sonoporation yielded significantly enhanced and prolonged T1 MRI contrast and increased intratumoural accumulation of both agents. These findings establish microbubble-free sonoporation as an effective, controllable technique for intracellular delivery, with the added benefit of MRI-based monitoring. This strategy addresses key limitations of microbubble-based methods and holds strong potential for image-guided therapeutic applications in solid tumors.

**Graphical abstract:**

**Figure:**
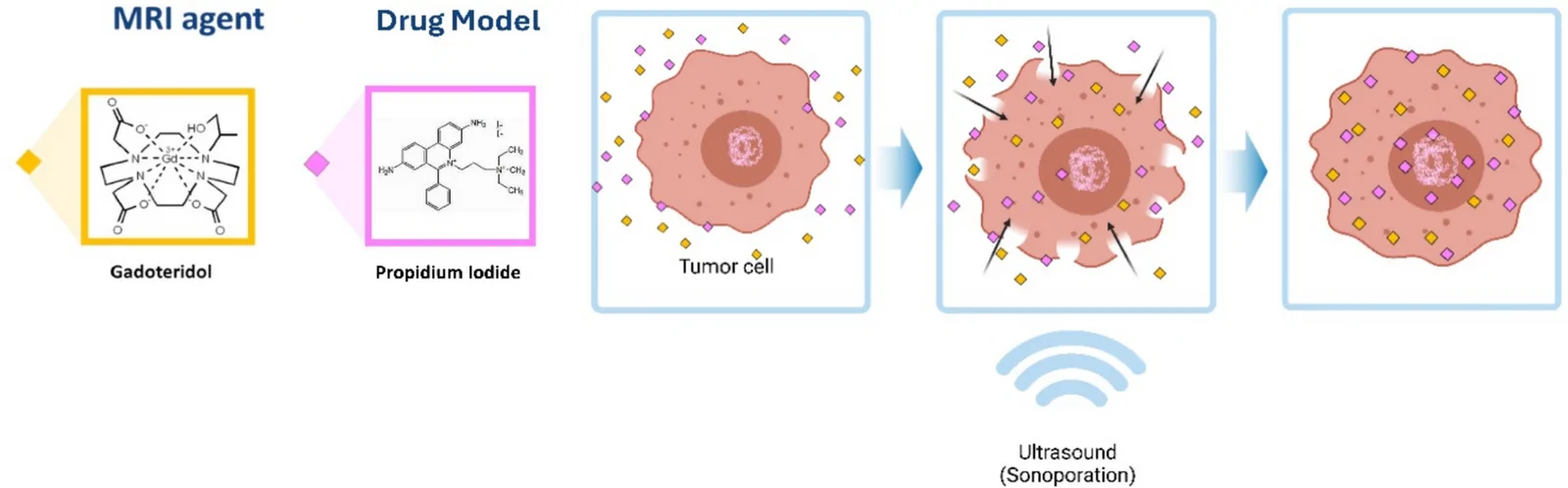

## Introduction

Ultrasound (US) stimulation is a widely accessible, cost-effective, minimally invasive, and safe technique for inducing molecular responses in vivo with precise spatial and temporal control. These capabilities support the development of advanced diagnostic and therapeutic strategies. Through its thermal and mechanical effects, US can be effectively exploited to enhance the delivery of bioactive compounds to target tissues.

Ultrasonic waves are highly versatile and defined by multiple tuneable parameters that govern their biological effects, including carrier frequency, mode of application (focused/unfocused, pulsed/continuous), and acoustic pressure. These parameters critically influence the balance between thermal and mechanical energies, which is essential for eliciting specific biological responses. Among the most promising biomedical applications of US is drug delivery, particularly through the transient permeabilization of cell membranes, commonly referred to as sonoporation (SNP), to promote site-specific accumulation of therapeutic agents.

To date, most sonoporation strategies rely on the administration of exogenous microbubbles, which serve as acoustic amplifiers during US exposure [1]. These microbubbles facilitate localised energy transfer via mechanisms such as inertial cavitation, stable cavitation, and bubble oscillation or translation [2–5]. However, their clinical use is limited by several drawbacks, including potential tissue damage, rapid clearance from circulation, and increased cost [6–10]. As a result, there is increasing interest in developing microbubble-free sonoporation techniques.

Mechanically driven, bubble-free sonoporation may be achieved through membrane deformation induced by acoustic radiation forces and shear stress from acoustic streaming [11–13], although the underlying biophysical mechanisms remain poorly understood. Notably, much of the recent research in this area has been conducted in microfluidic systems, which pose challenges for translation to in vivo conditions [14].

Another proposed mechanism involves the activation or cavitation of endogenous bubbles present within biological tissues [15–17], requiring stimulation within a defined Mechanical Index (MI) range (0.2–1.9) [18], and thus careful tuning of US frequency and pressure.

To date, only a limited number of studies have demonstrated successful sonoporation in biological environments without exogenous microbubbles [19–21], and most have focused on sonophoresis, ultrasound-enhanced transdermal absorption, rather than intracellular uptake. Moreover, the distinction between intracellular and intercellular transport across biological barriers remains underexplored.

A notable exception is the work by Chettab et al*.*, who used high-intensity focused US (MI = 3) to achieve intracellular delivery of doxorubicin via *in situ* cavitation [22].

**Fig. 1:**
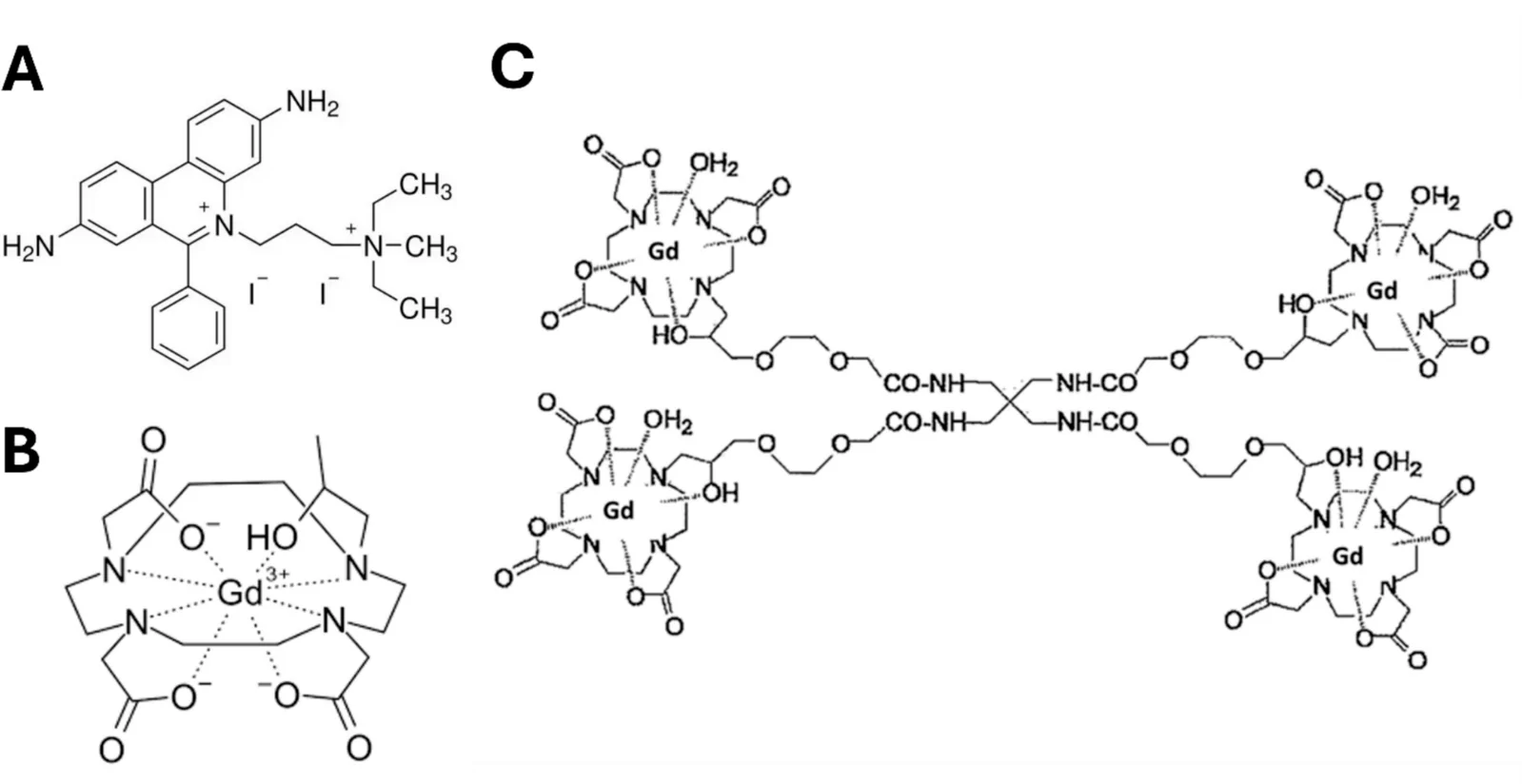
**A** Propidium Iodide (PI) **B**: Gadoteridol. **C**: Tetrameric version of Gadoteridol (Gado_4_)

Within this context, the present study investigates, for the first time to our knowledge, the use of non-focused, low-intensity US stimulation (MI = 0.25) to induce intracellular delivery of a model hydrophilic compound. We employed propidium iodide (PI, Fig. 1A) as a fluorescent marker for membrane permeabilisation and co-delivered the clinically approved MRI contrast agent Gadoteridol (Fig. 1B) to enable non-invasive in vivo monitoring of US-triggered molecular delivery.

## Materials and methods

### Chemicals and cells

Gadoteridol (Gd-HPDO3A) was kindly provided by Bracco Imaging (Colleretto Giacosa, Italy). Propidium Iodide (PI) was purchased from Sigma-Aldrich (St. Louis, USA). A tetrameric version of Gadoteridol (Gado_4_) (Fig. 1C) was synthesised as previously reported [23]. Human ovarian cancer (A2780), human myelogenous leukemia (K562), and murine macrophage (J774A.1) cell lines were purchased from American Type Culture Collection (ATCC, Manassas, VA, USA). TS/A murine breast cancer cells were derived at the University of Torino from a spontaneous mammary adenocarcinoma that emerged in a retired breeder BALB/c [24]. All cell lines were incubated at 37 °C in a humidified atmosphere containing 5% CO_2_. TS/A, A2780, and K562 were cultured in Roswell Park Memorial Institute (RPMI) 1640 Medium, whereas J774A.1 cells required Dulbecco's modified Eagle's medium (DMEM). All media were supplemented with 10% (v/v) of heat-inactivated Fetal Bovine Serum (FBS), 2 mM of L-glutamine, 100 IU/mL of penicillin, and 100 µg/mL of streptomycin. Cell lines were tested periodically to assess the absence of mycoplasma contamination using a biochemical assay MycoAlert™ Mycoplasma Detection Kit (Lonza, Verviers, Belgium). TS/A and A2780 cells, when reached 80% confluence, were harvested with a mixture of 170 IU/mL trypsin and 0.02% (m/v) ethylenediaminetetraacetic acid (EDTA) in sterile balanced salt solution and seeded in Nunc™ EasYDish™ dishes at density of *ca.* 1.5 × 10^4^ cells/cm^2^ and 3 × 10^4^ cells/cm^2^, respectively. K562 cells were grown in suspension and were subcultured at 1 × 10^6^ cells/mL and seeded in Nunc™ Cell Culture Treated EasYFlasks™ at density of 1 × 10^5^ cells/mL. J774A.1 cells at 80% confluency were gently detached using cell scraper (Corning Inc., Corning, NY) and seeded in Nunc™ Cell Culture Treated EasYFlasks™ at density of 2 × 10^4^ cells/mL. Dishes and flasks were obtained from Thermo Fisher Scientific (Massachusetts, USA). Culture media, FBS, L-glutamine, penicillin–streptomycin mixture and trypsin/EDTA mixture were all purchased from Lonza (Lonza, Verviers, Belgium).

### Ultrasound apparatus

The ultrasound apparatus consisted of the waveform generator KEYSIGHT 33500 B Series (Keysight technologies Ldt., Santa Rosa, CA, USA), a broadband RF power amplifier (Electronics & Innovation, Ltd., Rochester, NY, USA), an attenuator (Weinschel Associates Model WA23-3–34, Gaithersburg, MD, USA) and a piezoelectric ceramic flat disc transducer generating 1 MHz waves (Precision Acoustics Ltd, Dorset, UK). The transducer calibration was performed by Precision Acoustics Ltd and was based upon the sinusoidal burst technique as described in ASTM E1065-99 (2003) Standard Guide for Evaluating Characteristics of Ultrasonic Search Units. The acoustic pressure was measured at different voltages by Precision Acoustics Ltd. The calibration straight line was used to interpolate and obtain the acoustic pressure from the voltage. The MI was calculated from peak rarefactional pressure divided by the square root of the ultrasound frequency [25].

### In vitro cell labelling

For each labelling experiment, K562 cells were first harvested, counted, and pelleted by centrifugation (2.5 × 10⁶ cells, 200 × g). The pellet was gently resuspended in an isotonic solution containing the contrast agent and/or the model drug at the desired concentration, ensuring homogeneous exposure of all cells to the molecular probes. The resulting cell suspension was then transferred into the dedicated sample holder of a patented ultrasound-compatible plate system [26]. Ultrasound stimulation was performed using a piezoelectric transducer operating at 1 MHz in pulsed mode. The system allowed precise control of the acoustic waveform, including duty cycle, pulse repetition frequency, and voltage amplitude, enabling accurate definition of the applied acoustic pressure. Cells were exposed to ultrasound for 1 min under the optimised conditions described in the Results section. Immediately after insonation, samples were incubated at 4 °C for 30 min to allow complete membrane resealing while limiting active transport processes that could interfere with quantification of intracellular content. Cells were then extensively washed with PBS to remove extracellular residues of the contrast agent or model drug and finally resuspended in 100 μL of PBS for downstream analyses.

Control samples underwent an identical preparation workflow, including centrifugation, resuspension in the labelling solution, and incubation, but without exposure to ultrasound. This ensured that any observed intracellular uptake could be attributed specifically to the sonoporation stimulus rather than passive diffusion or handling-related artefacts.

For comparison with alternative cell-permeabilisation techniques, pinocytosis, electroporation, and hypotonic swelling were performed following previously established and validated protocols [23]. Briefly, cells were incubated with the corresponding chemical agent (100 mM) for 18 h at 37 °C for pinocytosis; electroporation was conducted using controlled electrical pulses optimised for K562 cells; and hypotonic swelling was induced by transient exposure to hypo-osmotic medium, followed by restoration of isotonic conditions. All conditions were processed in parallel to allow direct comparison of viability, uptake efficiency, and cellular stress.

### Relaxometric measurements of cell pellets and quantification of intracellular Gd

*Ca.* 25 million of cells were centrifuged (200 g rcf) for 15 min at 4 °C. Water protons longitudinal relaxation rate (R_1obs_) of the resulting pellets was measured by a Stelar SpinMaster Spectrometer (Stelar Snc, Pavia, Italy) at 0.5 T using the inversion recovery pulse sequence. Temperature control was performed through a STELAR VTC-91 airflow heater.

The amount of internalized Gd per cell was determined by relaxometry [27, 28]. Briefly, cells were lysed by sonication with Bandelin Sonopuls HD2070 Sonicator (20 kHZ, 21 W for 30 s). Lysates were mineralised by adding HCl (37%) (1:1 v/v) and maintained overnight at 120 °C to fully release the Gd(III) ion from the complex. Water protons longitudinal relaxation rate (R_1obs_) of the solution was measured at 0.5 T at 25 °C with a Stelar SpinMaster Relaxometer (Pavia, Italy), and the Gd(III) concentration was determined from the relaxivity value (13.7 mM^−1^ s^−1^) of the metal ion as follows:

$$[\mathrm{Gd}] = ({\mathrm{R}1}_{\mathrm{obs}}-0.5)/13.7$$

The cells number was determined as described above. Gd(III) was also quantified by Inductively Coupled Mass Spectrometry (ICP-MS, Element2, Thermo-Finnigan, Milano, Italy), after the mineralisation of the sample with HNO_3_ (37%) followed by microwave digestion (Milestone, Bergamo, Italy).

### MRI experiments on cell pellets

Cells were collected by centrifugation, suspended in 50 μl of PBS and placed into glass capillaries. After centrifugation (5 min at 200 × g), the capillaries were fixed in an agar support for in vitro MRI experiments. T_1_-weighted MR images were acquired at 7 T using a Bruker Avance 300 spectrometer (Bruker, Milano, Italy) equipped with a micro imaging probe. T_1_ values were measured by using a Saturation Recovery Spin Echo (SR-SE) sequence.

### Cell viability and proliferation tests

Cells viability was tested before and after the sonoporation treatment by the Trypan Blue Exclusion Test using Bradford assay for protein quantification. A standard for the Bradford assay was prepared using bovine serum albumin and a standard curve was set for every cell line to estimate the cell number. The viability was calculated as the percentage between the number of viable cells before (Nbt) and after (Npt) sonoporation. To assess proliferation, cells were counted, seeded at the specific density for every cell line, and maintained at 37 °C in humidified atmosphere containing 5% CO_2_. Cells were detached 24 h, 48 h, and 72 h after treatment and counted using the Trypan Blue Exclusion Test. The proliferation was calculated as the percentage of ratio between the number of viable cells immediately after the treatment (N_P0_) and the number of cells at each time point (N_P_), (N_P_/N_P0_*100).

### Quantification of intracellular PI

To determine the content of the cell internalised Propidium Iodide, cells were lysed by sonication with Bandelin Sonopuls Sonicator HD2070 (20 kHZ, 21 W for 30 s). The amount of PI was assessed spectrofluorometrically (λexc = 532 nm; λem = 671 nm) and calculated using a calibration line obtained using a set of standard samples of known concentration of PI. The cell number was determined using Trypan Blue Exclusion Test and Bradford assay as described above.

### Reactive oxygen species (ROS) production

To assess ROS production induced by US treatment, sonoporated cells were incubated with 2′,7′-dichlorofluorescein diacetate (DCF-DA) at a final concentration of 5 µM in PBS for 30 min at 37 °C [29, 30]. Following incubation, cells were washed with PBS, and fluorescence intensity was measured using a spectrofluorometer (λexc = 493 nm, λem = 522 nm). ROS levels were expressed as the fluorescence intensity of treated cells, normalised to cell number, relative to control cells. Results are presented as a percentage of the control (treated/control × 100).

### Animal model

All in vivo experiments were conducted in accordance with the European Directive 2010/63/EU for the protection of animals used for scientific purposes. The animal protocol was approved by the Italian Ministry of Health (Authorization No. 628/2018-PR). Mice had access to food and water ad libitum. 7-week-old athymic nude female mice (Envigo, Milano, Italy) were inoculated subcutaneously in the flank with 1 × 10^6^ K562 cells suspended in 100 µL of PBS. The tumour reached an adequate volume after 7 days. Animals were randomly assigned to the ultrasound-treated and control groups based on comparable tumour size. The mice were maintained under specific pathogen-free conditions in the animal facility. Before every treatment and MRI imaging, they were anaesthetised with a mixture of 5 mg/Kg xylazine (Rompum, Bayer, Milano; Italy) and 20 mg/Kg tiletamine/zolazepam (Zoletil 100, Vibac; Milano, Italy) administered intramuscularly.

### In vivo MRI experiments

MR images were acquired on a 7 T MRI scanner (Bruker AV300 spectrometer) equipped with a microimaging probe to assess the T_1_ contrast in the tumour area. Animals were divided in two groups: in control group, mice were administered via catheter in tail vein with Gadoteridol (0.3 mmol/Kg) and PI (8 mg/Kg), whereas in the SNP group the mice tumours were sonoporated for 1 min immediately after the co-injection of the contrast agent and the drug model. T_1w_ images were acquired using the T_1_ Multi Slice Multi Echo sequence (TR = 250 ms, TE = 3.2 ms, 6 averages, matrix size = 128 × 128, slice thickness = 1 mm and FOV = 3 × 3 cm), anatomical T_2w_ images were acquired by Rapid Acquisition with Refocused Echoes sequence (TR = 2000 ms, TE = 3.4 ms, effective TE = 27.20 ms, 4 averages, matrix size = 128 × 128, slice thickness = 1 mm and FOV = 3 × 3 cm). The T_1_ contrast enhancement (T_1_^Enh^) was calculated from the Signal Intensity (SI) values measured post-injection of the contrast agent (SI^Post^) normalised to the corresponding value measured before the injection (SI^Pre^), applying the following expression:

$${\mathrm{T}1}^{\mathrm{Ehn\%}}=[({\mathrm{SI}}^{\mathrm{Post}}-{\mathrm{SI}}^{\mathrm{Pre}})/{\mathrm{SI}}^{\mathrm{Pre}}]\text{ x}100$$

### Determination of intratumour Gd and PI content

To assess the amount of Gd accumulated in the tumour and muscles, harvested tissues were mineralised adding 1 mL of HNO_3_ (70%) and then heated by a microwave digestion system (Milestone, Bergamo, Italy) at 160° for 40 min. After mineralization, ultrapure water was added and samples were analysed by ICP-MS (Element2, Thermo-Finnigan, Milano, Italy). For PI quantification, tumours were harvested and homogenised on ice in Triton X-100 (1:100 v/v) (Homogeniser OV5; VELP Scientifica, Usmate Velate, Italy), sonicated on ice (Bandelin Sonopuls Sonicator HD2070, 20 kHZ, 21 W) for 30 s and centrifuged (10000 g) for 10 min. The fluorescence intensity was measured (FluoroMax-4, Horiba Jobin Yvon, Edison,USA) and the PI amount was calculated using a tissue-specific calibration line obtained using a set of standard and then normalised to the tumour weight.

### Ex vivo confocal fluorescence microscopy

Animals were divided in two groups: in control group, mice were administered via catheter in tail vein with PI (8 mg/Kg), whereas in the SNP group the mice tumours were sonoporated for 1 min immediately after the co-injection of the contrast agent and the drug model. After 30 min mice were sacrificed and tumors were excised, embedded in OCT, immediatelly frozen in liquid nitrogen, and then stored at 80 °C until further processing. 10 μm cryosections, obtained at different depth levels of the tumor.

Tumor slices were analysed with Laser Scanning Confocal Microscopy (Leica TCS SP5, Leica Microsystem Srl.). Experiments were carried out using dry lens. PI was visualized in the red channel (λex535 nm; λem617 nm). Hoescht dye (Sigma-Aldrich) was added for nuclear staining and visualized in the blue channel (λex358 nm; λem 461 nm).

### Statistical analysis

Results are representative of almost three technical replicates per experiment, unless otherwise stated. A statistical analysis using the t-student test was performed on each test series to determine if groups were statistically different. One-way ANOVA with Tukey’s post-hoc was performed to compare multiple groups. Two-way Analysis of Variance (ANOVA) with Bonferroni post-hoc was applied for multiple comparisons to identify specific group differences.

## Results and discussion

### In vitro sonoporation setup and optimization

In the initial phase of the work, the focus was directed towards optimising the ultrasound parameters to achieve high cell viability (exceeding 80%) and effective internalisation of the MRI agent. A critical factor in cell viability and proliferation after the sonoporation with microbubbles seems to be the size of the pores formed during ultrasound treatment [31, 32]. When these pores become excessively large (around 100 nm), there is a propensity for the efflux of certain molecules from cells, thus negatively affecting cell viability and function [33, 34].

**Fig. 2:**
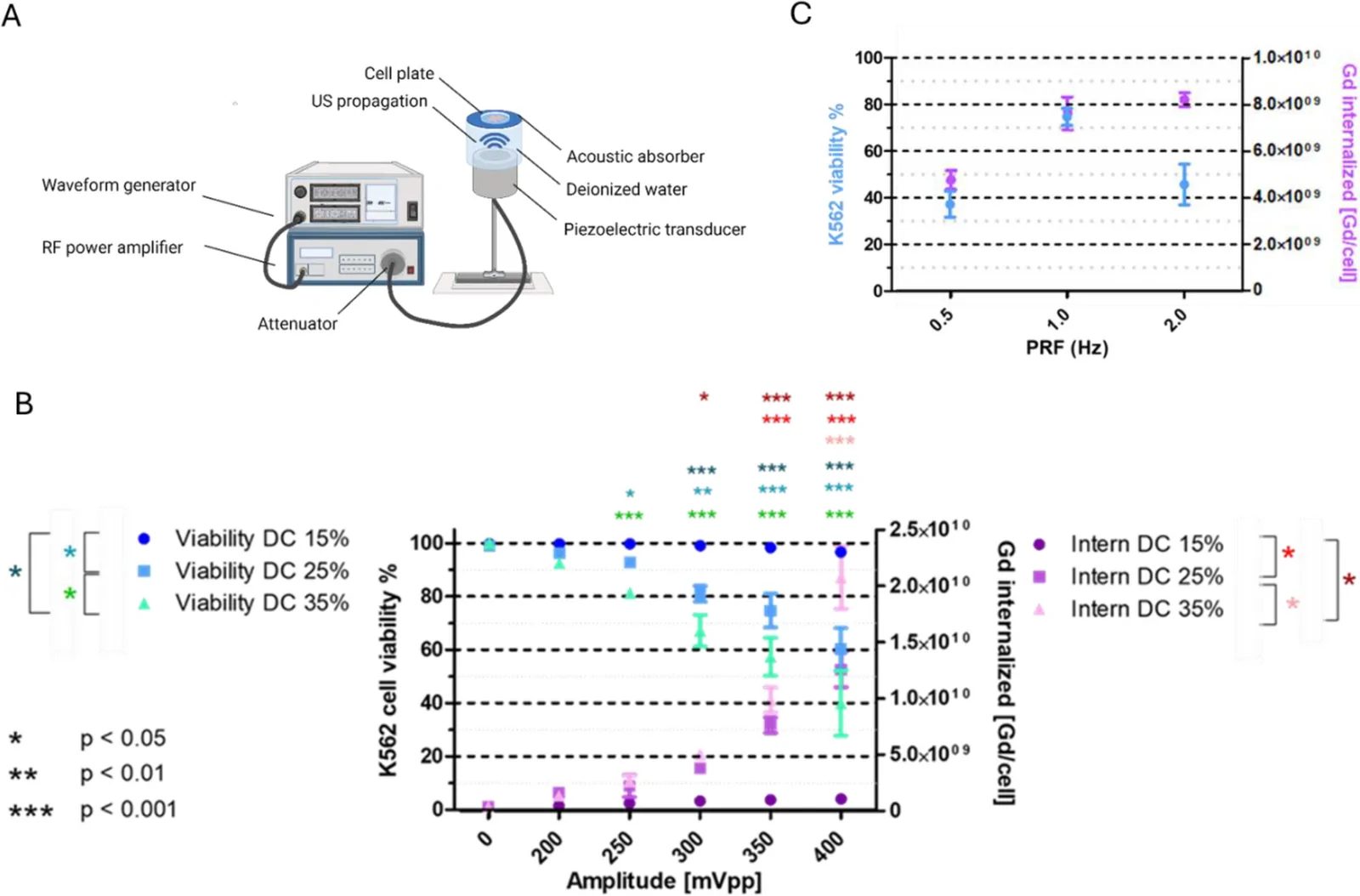
Optimisation of sonoporation setup. **A**) The ultrasound system is composed of the waveform generator, a broadband RF power amplifier, an attenuator, and a piezoelectric ceramic flat disc transducer generating 1 MHz waves. Between the transducer and the cell plate was placed a spacer of 0.07 m of both distilled and deionized water. Cells were placed in a cell plate. An acoustic absorber was placed on the water container to avoid unwanted ultrasonic reflection effects. **B**) Dependence of cell viability and uptake of Gadoteridol by K562 cells as a function of AP and DC (PRF 1 Hz). Viability was evaluated after sonoporation, varying the duty cycle (15% solid blue circle, 25% solid light blue square, and 35% solid heavenly triangle, an acoustic pressure of 0 corresponds to the condition without ultrasound exposure). Differences between the viability groups, analyzed as a function of Acoustic Pressure (AP) and Duty Cycle (DC), were assessed using a Two-way Analysis of Variance (ANOVA). Following a significant ANOVA result, a Bonferroni Post-Hoc Test was applied for multiple comparisons to identify specific group differences. The uptake of Gadoteridol was evaluated concurrently, varying the duty cycle (15% solid dark purple circle, 25% solid purple square, 35% solid pink triangle). The data reported in the graph are the average of three independent measurements. **C**) Dependence of cell viability (solid blue circle) and uptake of Gadoteridol (solid purple circle) by K562 cells as a function of PRF. DC 25% and AP 0.25 MPa parameters were fixed. The data reported in the graph are the average of three independent measurements. The data represent an average of 3 replicates

The sonoporation protocol was optimised using K562 cells incubated in an isotonic solution containing 60 mM Gadoteridol. Gadoteridol, an FDA-approved, clinically used MRI contrast agent with well-defined pharmacokinetics and high aqueous solubility, was intentionally selected as a model compound to evaluate ultrasound-mediated permeabilization without introducing formulation-dependent variables. The insonation system (Fig. 2A) consisted of a waveform generator, a broadband RF power amplifier, an attenuator, and a piezoelectric flat-disc transducer operating at 1 MHz. Efficient acoustic coupling between the transducer and the patented cell plate [26] was achieved by interposing a 7 cm layer of deionised, degassed water.

Cellular uptake efficiency was evaluated as a function of three parameters:

i) Acoustic Pressure (AP), modulated by adjusting the generator voltage;
ii) Duty Cycle (DC) of the pulsed US stimulation;
iii) Pulse Repetition Frequency (PRF).

The goal was to determine the optimal combination of parameters to achieve Gadoteridol internalisation > 7 × 10^9^ Gd ions/cell, while maintaining cell viability > 70%.

To evaluate the impact and interdependence of the two sonoporation parameters, Acoustic Pressure (MPa) and pulsed US stimulation (Duty Cycle, DC), on Gadoteridol uptake, a Two-way ANOVA was performed. The analysis demonstrates that uptake is significantly and interdependently influenced by both Acoustic Pressure and Duty Cycle (P < 0.0001).

To assess the significant differences between the mean internalized Gd measurements across the three Duty Cycle conditions (15%, 25%, 35%) at each Acoustic Pressure level (0 − 0.26 MPa), the Bonferroni post-hoc test was used.

Observation of the results revealed a clear and marked dependence of Gadoteridol uptake on the sonoporation parameters.

Uptake increases with increasing Duty Cycle (DC) and increases even more significantly with increasing Acoustic Pressure (AP). The Bonferroni post-hoc analysis supported these observations, confirming that statistically significant differences (P < 0.05) between the DC groups manifest exclusively at the highest Acoustic Pressures (0.24,0.25,0.26 MPa).

Specifically, at AP 0.26 MPa, all differences between the DC groups were highly significant (P < 0.001), with the DC 35% yielding significantly higher uptake compared to the other two conditions. Similarly, at AP 0.25 MPa, DC 35% and DC 25% showed significantly higher uptake compared to DC 15%. At AP 0.24 MPa, DC 35% was significantly superior to DC 15%. This evidence identifies the most intense parameter combination as generating the maximum uptake: AP 0.26 MPa and DC 35%, with a mean uptake of 2.077 × 10^10^. However, cell viability must also be considered: the viability analysis showed that this maximum-uptake condition resulted in a severe loss of cell viability (39.92%), which is below the acceptable clinical threshold.

The Two-way ANOVA confirmed the significant and interactive effect of the parameters on cell viability (P < 0.0001). Using the percentage of total variation explained as a measure of influential 'strength,' Acoustic Pressure (AP) was found to be the main source of variation (41.78% of total variability), followed by the Duty Cycle (DC) (33.93%), with the interaction accounting for 20.22%. Accordingly, while the AP exerted the greatest marginal influence on the system, the viability reduction itself followed a pattern: viability decreased as AP increased, but this effect was observed to be even more drastic with increasing DC.

The specific condition of AP 0.25 and DC 25% yielded a high cellular uptake of 7.63 × 10^9^, while successfully preserving cell viability at an acceptable level of 74.69%(± 6.34) (Fig. 2). A viability loss below 30% is considered acceptable in an oncological context, where moderate cell mortality does not compromise the intended therapeutic applicability [35].

The influence of PRF is shown in Fig. 2C. Lowering PRF to 0.5 Hz led to reductions in both uptake and viability, falling below the desired thresholds. Conversely, increasing PRF to 2 Hz slightly improved viability but reduced uptake.

Based on these findings, the following parameters were selected for all subsequent experiments:

$$\mathrm{AP}=0.25\text{ MPa }(\mathrm{MI}=0.25),\text{ DC}=25\mathrm{\%},\text{ and PRF}=1\text{ Hz}$$

### Cell proliferation, membrane resealing, and molecular uptake efficiency

To evaluate the impact of each component of the sonoporation protocol on cell growth, K562 cell proliferation was assessed following 1 min of treatment under four different conditions: i) cells incubated in PBS alone served as the baseline control; ii) incubation with Gadoteridol without ultrasound assessed whether the contrast agent affected proliferation; iii) ultrasound exposure in the absence of Gadoteridol allowed us to isolate the effect of acoustic stimulation on cell behaviour; iv) combining ultrasound with Gadoteridol represented the complete sonoporation treatment. This design enabled us to distinguish the individual and combined contributions of ultrasound and contrast agent exposure to post-treatment cell proliferation.

**Fig. 3:**
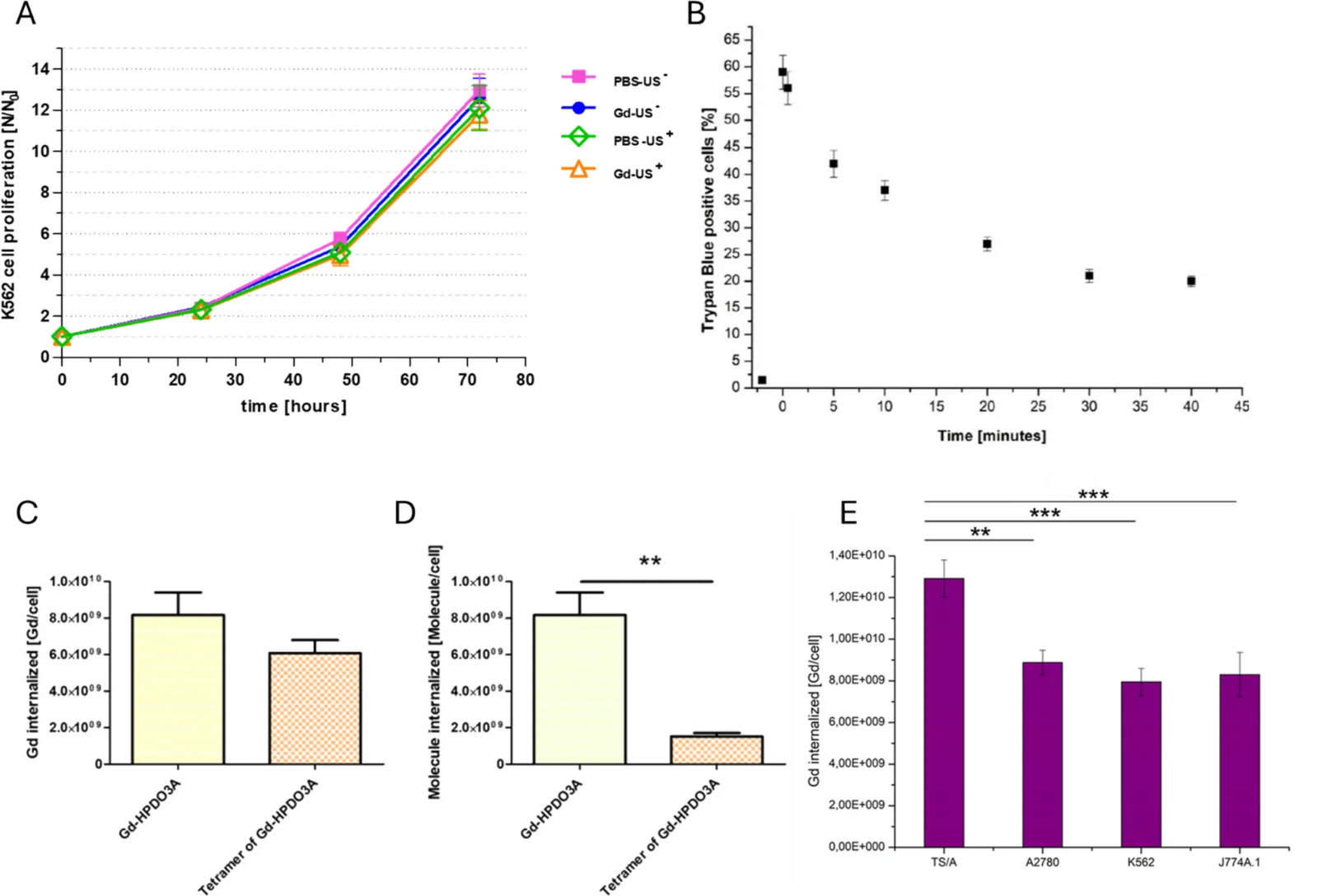
In vitro cellular uptake. **A**) K562 proliferation after incubation with PBS, Gadoteridol (Gd), with sonoporation. **B**) Internalization dynamics of Trypan Blue by K562 cells as a function of time after sonoporation stimulus. **C**) Gd internalized/cell on K562 cells incubated with 60 mM of Gadoteridol or Gado4 and then sonoporated. **D**) Molecular Gd-complexes internalized on K562 cells incubated with 60 mM of Gadoteridol or Gado4 and then sonoporated. Statistical analysis was performed using the T-student test. **E**) Cellular uptake of Gadoteridol (60 mM) by different cell lines after sonoporation. The data represent an average of three independent measurements. Error bars denote standard deviation (N = 3) (*, **, and *** respectively denote *p* < 0.05, *p* < 0.01, and *p* < 0.001) A statistical analysis using the t-student test was performed on each test series to determine if groups were statistically different. One-way ANOVA with Tukey’s post-hoc was performed to compare multiple groups

Proliferation was expressed as the ratio N_P_/N_P0_, where N_P_ is the number of viable cells at a given time point and N_P0_ is the number immediately after insonation (time 0). As shown in Fig. 3A, no significant differences in proliferation were observed across conditions, indicating that sonoporation did not impair cell growth under the experimental parameters used (P > 0.05).

To evaluate membrane resealing kinetics, Trypan Blue Exclusion was used to assess membrane integrity recovery post-US treatment. This assay relies on the principle that viable cells exclude trypan blue, while permeabilised or dead cells do not [36]. As shown in Fig. 3B, membrane integrity was restored within ~ 30 min. The residual uptake (~ 20%) likely corresponds to non-viable cells with irreversible membrane damage [37].

The impact of molecular size on uptake efficiency was assessed by comparing internalisation of Gadoteridol and its tetrameric analogue Gado4, both at 60 mM. No significant difference in Gd ions per cell was observed (P > 0.05, Fig. 3C). However, considering Gado4 contains four Gd ions per molecule, the uptake of Gadoteridol was ~ fourfold higher (P < 0.01, Fig. 3D), underscoring the influence of molecular size on sonoporation-mediated delivery.

To assess cell type dependency, uptake in K562 cells was compared with TS/A (murine breast cancer), A2780 (human ovarian cancer, adherent and suspension), and J774A.1 (murine macrophages). These cell lines were selected to represent a range of tumour and immune phenotypes, ensuring that the microbubble-free sonoporation protocol was evaluated across diverse membrane properties and translationally relevant biological contexts. All cell lines exhibited efficient uptake of Gadoteridol, with TS/A cells showing significantly higher internalisation (Fig. 3E).

The results presented herein demonstrate that the sonication parameters used facilitate the passage of small-sized molecules (such as gadoteridol and PI), making it difficult for the cellular internalisation of larger, but still in the sub-nm range, molecules like the tetrameric version of Gadoteridol (Gado4).

Furthermore, it has been demonstrated that using microbubbles, cell membrane pores close rapidly, typically within a minute [38], and such a swift closure may have a substantial impact on cell viability and function. In contrast, the proposed technique exhibits a much slower pore closure, taking approximately 30 min. Remarkably, despite this extended time frame for pore closure, cell viability and proliferation seem to remain unaffected.

The performance of microbubble-free sonoporation was evaluated across multiple cell lines, demonstrating its reliability in inducing transient membrane permeabilization. The slight variations observed among different cell types underscore the versatility of this technique and its potential for broad applicability in a wide range of solid tumours.

### Comparison of cell labelling methods

To assess the efficiency and safety of sonoporation for cell labelling, its performance was compared with three established techniques: pinocytosis (5 h of incubation), electroporation, and hypotonic swelling [23]. The evaluation focused on three key parameters: cell viability, Gadoteridol uptake, and reactive oxygen species (ROS) production, using K562 and J774A.1 cell lines.

**Fig. 4:**
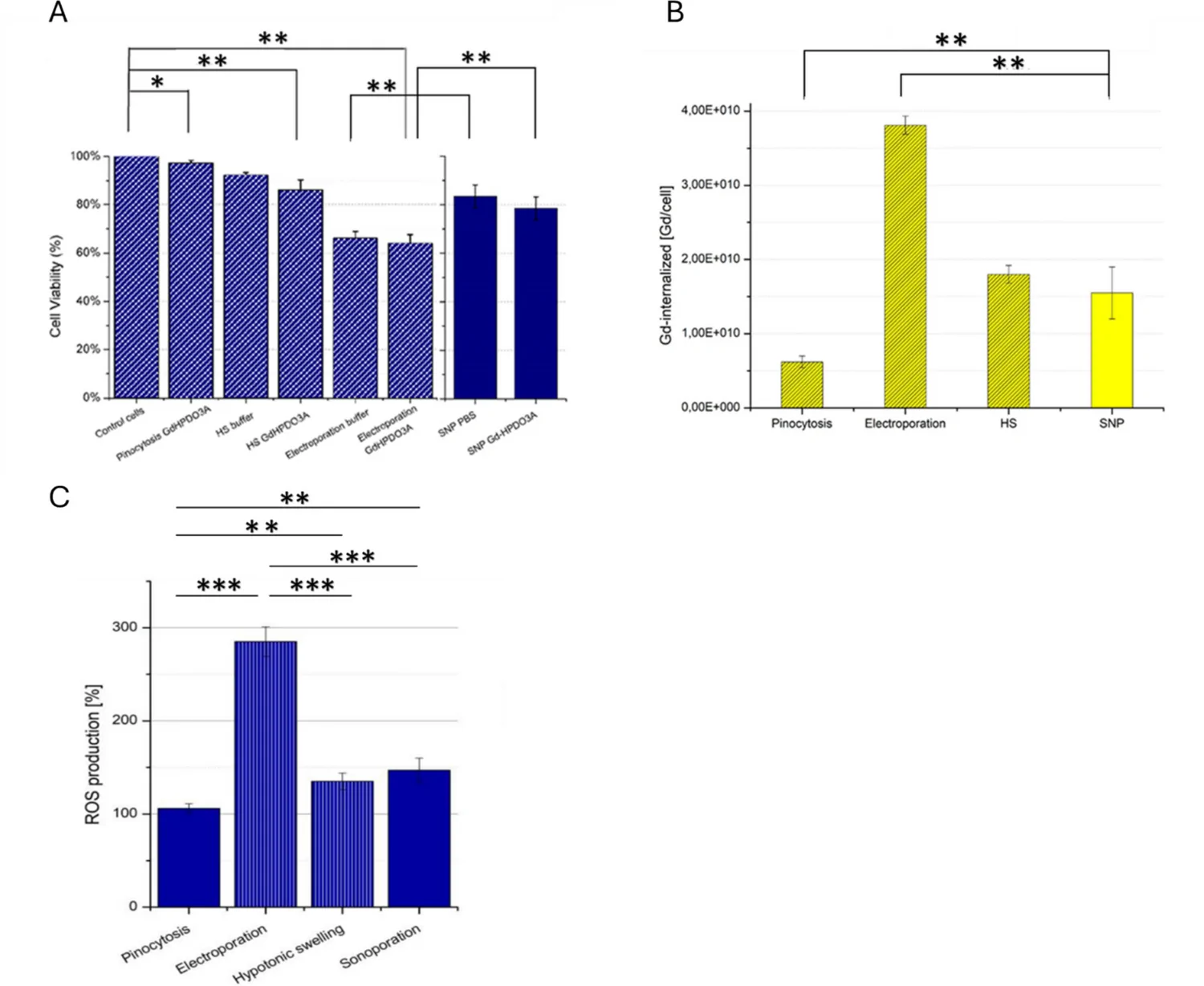
Comparison among different cell labelling methods. The data for pinocytosis, electroporation, and hypotonic swelling (HS) were taken from literature (patterned column) [23]. **A**) Cell viability of different labelling techniques on J774.A1 cells. **B**) Cellular uptake of Gadoteridol (60 mM) by different labelling methods **C**) % ROS production in J774A.1 cell. All data in the graphs are reported as mean values ± standard deviation of at least three independent experiments. (*, **, and *** respectively denote *p* < 0.05, *p* < 0.01, and *p* < 0.001) A statistical analysis using the t-student test was performed on each test series to determine if groups were statistically different. One-way ANOVA with Tukey’s post-hoc was performed to compare multiple groups

Cell viability (Fig. 4A shows data for J774A.1 cells) followed the trend: Pinocytosis (~ 100%) > Hypotonic swelling (~ 90%) > Sonoporation (~ 80%) > Electroporation (~ 70%).

In contrast, Gadoteridol uptake displayed the opposite trend (Fig. 4B): Electroporation > Sonoporation ≈ Hypotonic swelling > Pinocytosis.

ROS production, used as an indicator of cellular stress [39], was also analysed (Fig. 4C). As previously reported [23], electroporation induced the highest ROS levels, consistent with its lower viability. Pinocytosis did not significantly affect ROS levels, although it was also the least effective in terms of labelling efficiency. Both sonoporation and hypotonic swelling resulted in a modest increase in ROS, indicating a relatively mild oxidative stress response.

The cell viability following sonoporation without microbubbles appears to be comparable to that achieved through hypotonic swelling, a technique based on the hypo-osmotic shock treatment applied in vitro, but impractical for in vivo applications. Conversely, sonoporation without microbubbles appears to be promising for in vivo use. The overall uptake seems to be lower than that achieved with electroporation, but it remains significantly higher than simple pinocytosis. However, electroporation appears to induce noticeably greater damage in terms of reactive oxygen species production and subsequent impact on cell viability.

Overall, these results highlight sonoporation as a balanced technique, offering efficient intracellular delivery with acceptable viability and limited oxidative stress, positioning it as a promising alternative to more disruptive methods like electroporation.

### In vitro internalisation of gadoteridol and propidium iodide

To investigate the intracellular fate of the MRI cell-labelling agent following sonoporation, relaxometric analysis was performed. The primary internalisation pathway for Gd-based contrast agents is endocytosis [27, 40], which can limit the effectiveness of MRI detection. When the intracellular concentration of Gd-complexes exceeds ~ 2 × 10^9^ molecules/cell, endosomal entrapment leads to a significant reduction in observed longitudinal relaxivity. This occurs when the difference between the relaxation rates of water protons in the endosomal (R_1end_) and cytoplasmic (R_1cyt_) compartments, in the absence of exchange, exceeds the actual exchange rate, resulting in relaxivity quenching [41, 42]. Conversely, when Gd-complexes are distributed in the cytoplasm, this quenching effect is not observed, and relaxivity remains high.

To distinguish between these mechanisms, longitudinal relaxation rates were measured under two conditions:

i) cells incubated with Gadoteridol for 18 h at 37 °C at concentrations of 90, 100, and 120 mM;
ii) cells incubated for 1 min at the same concentrations, followed by sonoporation.

**Fig. 5:**
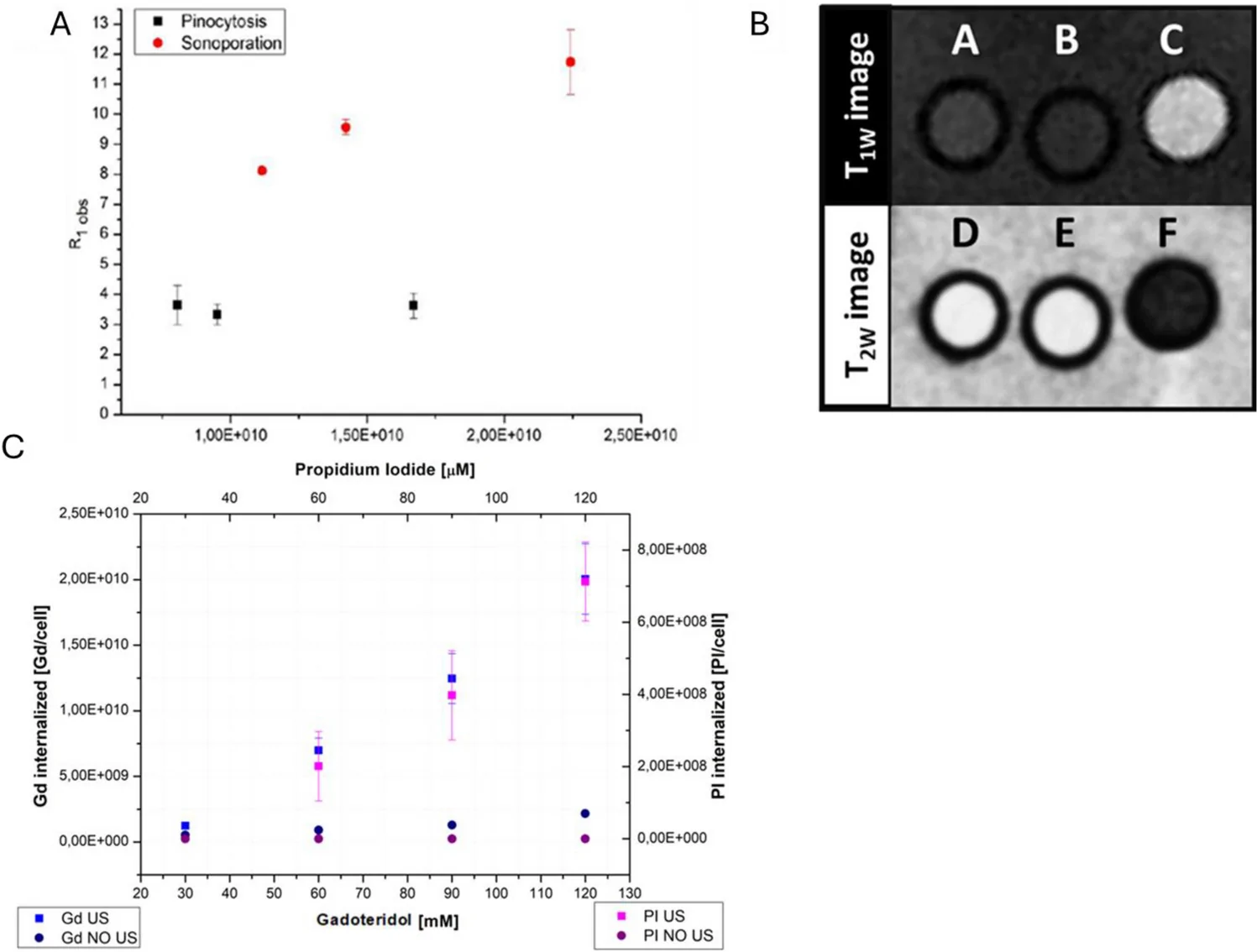
In vitro co-internalisation of gadoteridol and PI **A**) R1_obs_ measured in cells containing different amounts of Gd after sonoporation and pinocytosis B) T1w (**A-C**) and T2w (**D-F**) MR images of a phantom containing gadoteridol labeled-K562 cellular pellets. Cell pellets B and E were incubated with PBS, cell pellets A and D were labelled by pinocytosis and cell pellets C and F were labelled by sonoporation. The phantom contains the following samples: A) cells incubated with gadoteridol but not sonoporated, 2) cells in PBS sonoporated, 3) cells incubated with gadoteridol and sonoporated. C) Sonoporation-induced co-internalisation of gadoteridol and PI by K562 cells. All data in the graphs are reported as mean values ± standard deviation of at least three independent experiments

As shown in Fig. 5A, sonoporated cells exhibited significantly higher relaxivity and T_1_ MR contrast, suggesting that sonoporation enables cytoplasmic distribution of the MRI agent. These findings are consistent with a mechanism involving transient pore formation, although they do not exclude the possibility that endocytic pathways also contribute to the observed uptake.

To assess MRI detectability, T_1_-weighted and T_2_-weighted MR images of cells labelled via pinocytosis or sonoporation were acquired at 7 T. The T_1_-weighted image of sonoporated cells showed significantly greater contrast than those labelled by pinocytosis (Fig. 5B), with a similar enhancement in T_2_ contrast. These results confirm that sonoporation enables rapid and efficient cytoplasmic delivery, improving MRI visibility.

To further evaluate co-internalisation, K562 cells were treated with ultrasound in the presence of Gadoteridol and propidium iodide, using a fixed 1000:1 molar ratio. As shown in Fig. 5C, the uptake of both agents increased linearly with their concentration in the incubation medium. Control experiments without US exposure showed negligible passive uptake. The linear correlation indicates that gadoteridol and PI are internalised independently, without mutual interference.

The intracellular delivery pathway induced by sonoporation was investigated through relaxometric analysis. While previous studies have reported that microbubble-assisted sonoporation can promote molecular uptake via endocytosis [43], our findings indicate that, in the absence of microbubbles, the process primarily involves the formation of transient membrane pores, enabling direct cytosolic entry of molecules.

This mechanism, which is shared by other cell-labelling techniques, such as electroporation and osmotic shock, has significant implications for MRI-based cell tracking. In particular, the ability of the contrast agent to generate T_1_ signal enhancement is markedly improved when it is not confined within intracellular vesicles.

Furthermore, post-sonoporation analyses revealed that the uptake efficiency of the MRI contrast agent closely mirrors that of the model drug. This correlation is especially relevant, as it suggests that the contrast agent can serve as a reliable surrogate marker for monitoring drug delivery, thereby enabling non-invasive tracking of therapeutic uptake in future in vivo applications.

### In vivo MRI evaluation of sonoporation-mediated delivery

**Fig. 6:**
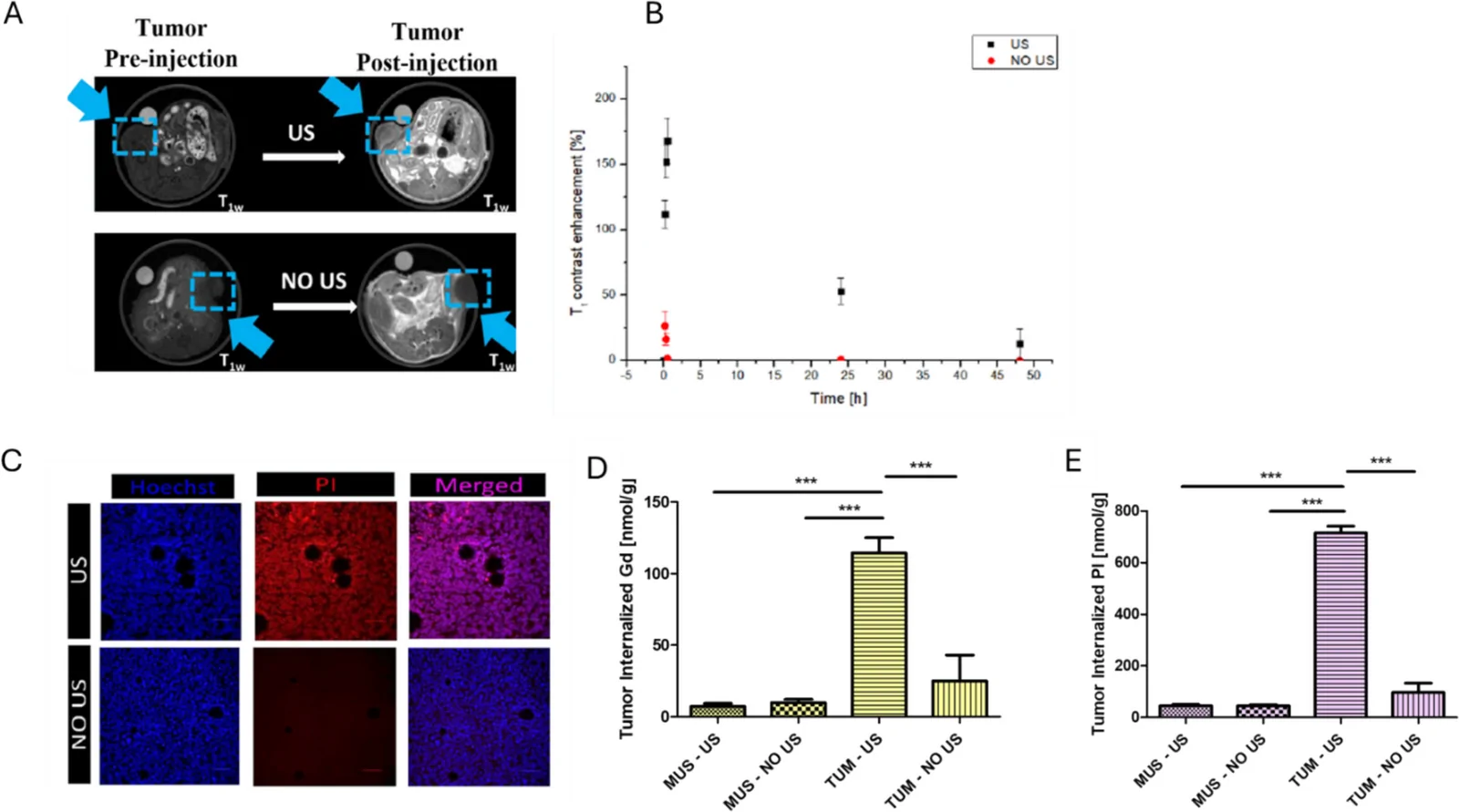
In vivo MRI evaluation of sonoporation-mediated delivery. **A**) T_1w_ MR images of representative mice of sonoporated and not stimulated (NO US) groups. Dashed squares localize the tumour before (left) and after (right) the co-injection of gadoteridol (0.3 mmol/kg) and PI (8 mg/kg). **B**) T_1_ contrast over time for the two groups. **C**) Confocal fluorescence images of K562 tumors explanted from mice of US-stimulated (US) and not stimulated (NO US) groups co-injected with gadoteridol (0.3 mmol/kg) and PI (8 mg/kg). Red signal was emitted by PI and blue signal by Hoechst dye staining cell nuclei. Tumours were explanted 30 min after the systemic co-administration of gadoteridol and PI and sonoporation. **D**) Ex vivo quantification of gadoteridol in the tumor and muscle of the sonoporated and non-sonoporated groups. E) Ex vivo quantification of propidium iodide in tumor and muscle of sonoporated (US) and not sonoporated (NO US) groups. All data in the graphs are reported as mean values ± standard deviation of at least three independent experiments. (*, **, and *** respectively denote *p* < 0.05, *p* < 0.01, and *p* < 0.001). Statistical analysis was performed using one-way analysis of variance (ANOVA) with Tukey’s post analysis

In vivo MRI experiments were conducted on two groups of mice (n = 3), each subcutaneously inoculated with K562 cells. Both groups received intravenous injections of gadoteridol (0.3 mmol/kg) and propidium iodide (PI, 0.012 mmol/kg); however, only one group underwent local ultrasound stimulation (sonoporation) at the tumour site immediately after injection. T₁-weighted (T_1w_) and T₂-weighted (T_2w_) MR images were acquired pre- and post-treatment to assess contrast enhancement and tumour localisation (Fig. 6A).

As neither gadoteridol nor PI possesses intrinsic cell-penetrating properties, the persistence of T_1_ contrast after systemic washout indicates agent retention within the tumour, likely via intracellular accumulation. T_1w_ MRI over 48 h showed significantly higher and longer-lasting contrast in sonoporated tumours compared to controls. In untreated mice, contrast intensity declined rapidly, returning to baseline within 30 min post-injection (Fig. 6B).

To confirm intracellular delivery, confocal fluorescence microscopy was performed on tumour sections. Images (Fig. 6C) showed strong PI-associated red fluorescence in sonoporated tumours, co-localised with Hoechst-stained nuclei, consistent with intracellular uptake. In contrast, non-sonoporated tumours exhibited only minimal fluorescence.

Quantitative analysis of Gd and PI uptake was performed on excised tumour and muscle tissues (the latter serving as internal control), collected 30 min post-treatment. Gd content was quantified by ICP-MS after tissue mineralization. Sonoporated tumours showed significantly higher Gd levels compared to both control tumours and muscle (Fig. 6D), consistent with enhanced uptake facilitated by sonoporation and the leaky vasculature of solid tumours. Similarly, PI levels, measured by spectrofluorimetry, mirrored the distribution pattern of gadoteridol (Fig. 6E), confirming co-accumulation of the model drug.

Hence, following the local application of ultrasound to the tumour site in a murine model, immediately after intravenous co-administration of the MRI contrast agent and the drug model, MRI acquisitions revealed a clear and sustained enhancement of T_1_ contrast within the tumour. This effect, absent in non-sonoporated control animals, indicates substantial tumour accumulation of gadoteridol. Ex vivo confocal fluorescence microscopy performed on excised tumours further confirmed the efficient intratumour accumulation of the drug model in the sonoporated group.

Finally, quantitative analysis showed a 4.5-fold increase in gadoteridol content and a 7.4-fold increase in drug model uptake in treated tumours compared to controls. These values are consistent with those reported in previous studies using microbubble-assisted sonoporation [43–45], demonstrating that microbubble-free sonoporation achieves comparable delivery efficiency.

## Conclusions

The proposed microbubble-free sonoporation system demonstrates high potential for achieving transient cell membrane permeabilisation in vitro, with reduced cytotoxicity and minimal impact on cell proliferation compared to microbubble-based methods. This improved biocompatibility is likely attributable to the formation of smaller, transient pores, which close more gradually. Such pore dynamics allow for the direct cytosolic entry of molecules, bypassing endosomal entrapment and degradation typically associated with endocytic uptake.

However, this mechanism currently limits the system's applicability to the internalisation of small molecules, and may not be suitable for gene delivery applications. Despite this limitation, the ability to co-deliver imaging contrast agents presents a valuable opportunity for image-guided ultrasound therapy, a promising approach to enhance and monitor the efficacy of chemotherapy while enabling the prediction of therapeutic outcomes [46, 47].

Moreover, in vivo experiments confirmed the system’s high efficiency in promoting intratumour accumulation of imaging and model therapeutic agents, highlighting its translational potential in drug delivery applications.

Collectively, our findings support microbubble-free mechanical sonoporation as a versatile, controllable, and effective platform for MRI-guided drug delivery in solid tumours. Future investigations will aim to expand its utility to therapeutic compounds and to integrate real-time imaging strategies, advancing its development toward clinical translation.

## Data Availability

The datasets generated during and/or analysed during the current study are available from the corresponding author on reasonable request.

## Declarations

All institutional and national guidelines for the care and use of laboratory animals were followed.
